# Receiver operating characteristics of impulse oscillometry parameters for predicting obstructive sleep apnea in preobese and obese snorers

**DOI:** 10.1186/s12890-016-0284-3

**Published:** 2016-08-22

**Authors:** Arikin Abdeyrim, Liang Tang, Arzugl Muhamat, Kelimu Abudeyrim, YongPing Zhang, NanFang Li, Yinchun Wang, Minghua Zhao

**Affiliations:** 1 Department of Otorhinolaryngology Head and Neck Surgery, The People’s Hospital of Xinjiang Uygur Autonomous Region, Ürümqi, China; 2 Postgraduate College of Xinjiang Medical University, Ürümqi, China; 3 Department of Respiration Medicine, Xinjiang Petroleum Administration MingYuan Works Hospital, Ürümqi, China; 4 Department of Hernia and Abdominal Wall Surgery & Minimally Invasive Surgery, The People’s Hospital of Xinjiang Uygur Autonomous Region, Tianchi Road No. 91 Tianshan District, Ürümqi, Xinjiang 830001 China; 5 Hypertension Diagnosis and Treatment Center, The People’s Hospital of Xinjiang Uygur Autonomous Region, Ürümqi, China; 6 Laboratory of sleep study, The People’s Hospital of Xinjiang Uygur Autonomous Region, Ürümqi, China; 7 Respiratory Function Test Department, The People’s Hospital of Xinjiang Uygur Autonomous Region, Ürümqi, China

**Keywords:** Respiratory function test, Receiver operating characteristics (ROC), Obstructive sleep apnea syndrome (OSAS)

## Abstract

**Background:**

Inability to maintain upper-airway patency during sleep is a cause of obstructive sleep apnea (OSA) and its sequelae. The associated syndrome (OSAS) is common in obese populations, currently, nocturnal polysomnography is the gold standard for diagnosing this conditions, but the diagnostic procedures are expensive and time-consuming. Therefore, identification of new markers of OSAS would be useful. This study aims to examine the receiver operating characteristics of impulse oscillometry (IOS) parameters for the prediction of OSAS in preobese and obese snoring patients.

**Methods:**

In total, 230 patients with normal spirometric values were included in this cross-sectional study. Full laboratory polysomnography was performed and IOS measurements were determined in sitting and supine positions to obtain respiratory impedance (Zrs), resistance (Rrs), and reactance (Xrs) parameters. The respiratory resistance at zero-frequency (Rrs0) was extrapolated by linear regression analysis of Rrs versus low-oscillatory-frequencies and its inverse, respiratory conductance (Grs), was calculated.

**Results:**

In both the sitting and supine positions Rrs0, Zrs, and Rrs at five oscillatory-frequencies (Hz) and Grs, the reciprocal of Zrs5 (Gz), and Xrs at 5 Hz all had significant positive or negative correlations with OSAS severity as defined by the Respiratory disturbance index (RDI). The correlation coefficients between Rrs0, Zrs5, Rrs5, Grs, Gz, Xrs5 measured in the supine and RDI were 0.425, 0.395, 0.378, −0.425, −0.395, and −0.517, respectively (all *p* < 0.001). The receiver operating characteristics curves showed that Xrs at 5 Hz (reactance) in the supine position was the best for predicting OSAS with a sensitivity of 73 % and specificity of 84 % at the optimal cut-off point of −0.23 (kPa s L^−1^). The other parameters also showed acceptable discriminating power. A logistic-regression model based on respiratory function abnormalities revealed that reactance combined with patient sex and lung volume yielded a specificity of 83.3 % with a sensitivity of 76.8 % for indicating OSAS.

**Conclusion:**

Respiratory resistance and reactance measured by IOS are abnormal in preobese and obese OSAS patients, and these parameters are moderate to closely correlated with OSAS severity. IOS might be a useful screening tool for detecting OSAS in clinic based populations.

## Background

Obstructive sleep apnea (OSA) is a common disorder characterized by a repetitive collapse of the pharyngeal airway during sleep. It manifests as reduced (hypopnea) or absent (apnea) airflow at the nose/mouth, despite ongoing inspiratory efforts, which is terminated by a transient arousal from sleep and restoration of upper-airway patency [1, 2]. Consequently, this pathophysiological process causes disturbances in blood gases and sleep structure and has also been associated with cardio-cerebrovascular complications [3–5].

Nocturnal polysomnography (PSG) in a sleep laboratory is the standard method for diagnosing OSA. However, PSG is expensive, time-consuming, and labor-intensive. There is a high prevalence of OSA and the associated syndrome (OSAS) in obese populations, yet few centers can afford to perform the diagnostic procedure on all patients presenting with OSA. In this context, simpler and less expensive tests are needed.

Even through physical upper-airway stenosis and increased pharyngeal airway collapsibility during expiration plays an important role in these patients, [6, 7] it is expected that the cycle of obstruction and restoration of upper-airway patency from arousal will be accompanied by large swings in intrathoracic pressure and changes in the mechanical properties of the respiratory system [2]. Van Noord et al. reported that respiratory resistance (Rrs) at low oscillatory-frequencies measured by the forced-oscillation technique (FOT) increased with a decrease in oscillatory frequency in patients with upper-airway obstruction; Also, this patients was showed a decrease in respiratory reactance (Xrs) with a increase of oscillatory frequency [8]. Zerah et al. demonstrated that Rrs increases linearly with a decrease of oscillatory frequency over the 4–16 Hz frequency range in obese subjects. This characteristic can be used to extrapolate total Rrs, that is, the zero-frequency resistance (Rrs0), which was found to increase with the level of obesity as a result of a reduction in lung volume [9]. Consequently, Zerah et al. analyzed Rrs data obtained in obese OSA patients with a sitting posture and back-extrapolated the regression line to 0 Hz to obtain the Rrs0 parameter, and its inverse–respiratory conductance (Grs) as well as specific Grs (sGrs) was calculated: the ratio of Grs over functional residual capacity (FRC), and those FOT parameters was found to be independent from their BMI associated with OSA severity [10]. Additionally, the parameter of sGrs was showed a high predictive value for OSAS diagnos, defined as the Apnea–Hypopnea Index (AHI) ≥15 [11]. These results suggest that the caliber of both pharyngeal airway and intrathoracic airways in obese OSA patients are commonly prone to collapse on the exhale, due primarily to decrease in lung volumes. The cross-sectional area of pharyngeal airway as well as peripheral airways are well known to varies considerably with alterations in lung volume. The lumen size in those structures are proved to a decrease when the end-expiratory lung volume (EELV) or FRC are artificially lowered, that either caused by negative expiratory pressure (NEP) or by positive extrathoracic pressure on exhalation, and manifests as expiratory flow limitation (EFL) in the both structures as well as airflow resistance are markedly increased [12–14]. These phenomena appear to be more pronounced in obese OSAS patients while adopt on the supine position. It has been demonstrated in obese subjects with and without OSA that lung volume, in term of EELV or FRC, would be a further decrease when the supine position is take on from a seated. Such a lung volume decrease may facilitate pharyngeal airway and intrathoracic airways to collapse or even closure, due to loss effects of caudal traction tension on both structures, and contribute to resistance increase in the airways.

Recently, we identified some parameters of impulse oscillometry (IOS) measured in obese OSA patients with sitting position, such as respiratory impedance (Zrs), Rrs, and Xrs at 5 Hz were significantly correlated with OSA severity as defined by AHI [15]. IOS, a type of FOT is used to determine the mechanical properties of the respiratory system during tidal breathing in recent years [16]. It is easy to perform, requires minimal subject cooperation, and the measurements can be recorded in any body position. More than twice the number of respiratory events in majority of the obese OSA population are occur in the supine position, [17, 18] the underlying mechanisms of it has been attributed to decrease in lung volume with a reduce favorable effects of caudal tracheal traction on pharyngeal airway [19]. It can be speculated from such evidence that the mechanical properties of the intrathoracic airways would also be implicated by the same mechanisms mentioned above when obese OSA patients sleeping in the supine. Hence, we hypothesize that parameters of IOS obtained in the supine could higher the capacity of IOS test in the seated position to differentiate between obese snoring persons and those more severely affected – namely, patients with OSAS.

The aim of this study was to develop a diagnostic tool, not requiring polysomnography, for identifying OSAS in snoring patients whose risk of OSAS is not too high. We have therefore undertaken a large prospective study to determine the optimal operating characteristics for the use of IOS as a screening tool for detecting OSAS in preobese and obese snoring patients.

## Methods

### Patient selection

In total, 249 preobese and obese subjects with a body mass index (BMI) greater than 25 kg/m^2^ and attending a sleep clinic for the first time for suspicion of OSAS due to snoring were eligible for the study. Exclusion criteria were a history of alcoholism, regular use of hypnotic medication, previous treatment for sleep apnea, a history and physical examination compatible with cardiopulmonary disease, and the presence of airway obstruction due to asthma or to chronic obstructive pulmonary disease (forced expiratory volume in 1 s [FEV_1_]/forced vital capacity [FVC] less than 80 % of predicted volume). Subjects with evidence of neuromuscular disease were also excluded.

### Sleep studies

Overnight sleep studies were performed in all participants, and consisted in full laboratory PSG including electroencephalography (C4-A1, C3-A2), right and left electrooculography, chin electromyography, oronasal airflow, thoracic and abdominal movements (inductive plethysmography bands) and oxygen saturation monitored via a finger probe. Respiratory events were defined as follows: [20] a nasal pressure drop to ≥ 30 % of baseline and associated with ≥ 3 % desaturation, lasting for at least 10 s was scored as hypopnea or associated with an EEG arousal was scored as respiratory effort-related arousals (RERAs). Absence of airflow on a nasal pressure transducer and < 10 % baseline fluctuations on a thermistor signal lasting for > 10 s, was scored as apnea. Respiratory disturbance index (RDI) was calculated by dividing the total apneas, hypopnea, RERAs by the total sleep time in hours, a RDI⩾15/h of sleep was used to define the presence of OSAS, [20] and was used as the gold standard in the evaluation of the operating characteristics of the IOS parameters obtained in the seated and the supine position to detect OSAS.

### Measurements

Pulmonary function and IOS tests were performed by experienced pneumologists aided by a technician, the morning before or after snoring subjects attended an overnight sleep study. All measurements and clinical evaluations were assessed blindly: pneumologists were unaware of the sleep status of the patient and, conversely, the physician who performed the polysomnography was not aware of the lung function test results.

### Spirometric and lung volume tests

Three maximal flow-volume loops were obtained in the seated position using a MasterScreen pneumotachograph (Jaeger® CareFusion, Germany), with the largest retained to calculate the FEV_1_/FVC ratio. Static lung volumes were determined after spirometry using the MasterScreen body plethysmograph (Jaeger®). Functional residual capacity (FRC) was measured while subjects gently breathed against the shutter at a rate of <1/s. Expiratory reserve volume (ERV) and inspiratory capacity (IC) were also measured during the same maneuver, and the total lung capacity (TLC) was calculated as FRC + IC. All values are reported as actual values.

### Mechanical properties of the respiratory system measured by IOS

IOS (Jaeger®) measurements were made while a technician supported the cheeks of subjects who were wearing nose-clips in the sitting position, and then in the supine position, fulfilling standard recommendations [21]. In short, the subjects were advised to tightly seal their lips around the mouthpiece while breathing quietly at FRC levels. Impulse signals originated from a generator at intervals of 0.2 s, and the rectangular pressure impulses are superimposed on airflow, which are feed to the airway during tidal breathing via a mouthpiece, after stable spontaneous volume and airflow were confirmed and a minimum of three consecutive measurements of >30 s were taken. As results of IOS measurement, we used the parameters of Zrs at 5 Hz (Zrs5), mean whole-breath values of Rrs and Xrs between 5 Hz and 35 Hz in 5 Hz increments (R5–R35 and X5–X35, respectively), and resonant frequency. Linear regression analysis of the Rrs over the frequency range between 5 and 15 Hz was used to calculate the intercept resistance, that is, the Rrs at 0 Hz (Rrs0). This is relates to the total resistance of the respiratory system, and is usually used as an index of airway obstruction. Grs was then calculated as the reciprocal of the intercept resistance (Grs = 1/Rrs0). In addition, the value of the Zrs5 yield by IOS is believed to be equivalent to Rrs0, and respiratory conductance was also calculated as the reciprocal of Zrs5 and expressed as Gz.

### Statistical analysis

All analyses were performed using SPSS software (IBM Corp, Armonk, NY, USA). Data are expressed as means ± standard deviation (SD). According to outcome measurements of overnight PSG the subjects were distributed into three groups: a RDI of ⩾15 per hour was classified as OSAS, a RDI of 5 or more, but fewer than 15 events/h was classified as mild–OSA, and the subjects with a RDI <5 per hour was classified as healthy snorer. Differences of anthropometry (age, height, weight, BMI), lung volume and function were assessed between healthy snorer and mild–OSA and OSAS patients using one-way ANOVA. Correlations between IOS measurements in both the positions (sitting and supine) and RDI and BMI were evaluated using Spearman’s rank correlation coefficients. The sensitivity and specificity of possible cut-off points for the parameters of IOS obtained in both positions, to discriminate between OSA patient (snorers and mild–OSA) and OSAS patients, were identified using a receiver operator characteristics (ROC) curve. This allowed the visualization of the true positive rate (sensitivity) as a function of the false positive rate (1 − specificity) for the different respiratory mechanical property parameter values, and was used to identify the cut-off value yielding the largest number of correctly classified patients. Next, to assess which of the IOS parameters in the supine that could best predict the probability of having a PSG positive result for OSAS, backward and forward stepwise logistic regressions were performed with lung volume measurements, the parameters of IOS, and anthropometry as explanatory variables, and with the presence of OSAS (RDI ⩾15) or absence of OSAS (RDI < 15): a binary (yes/no) outcome as the dependent variable. *P*-values less than 0.05 were considered statistically significant.

## Results

**Fig. 1:**
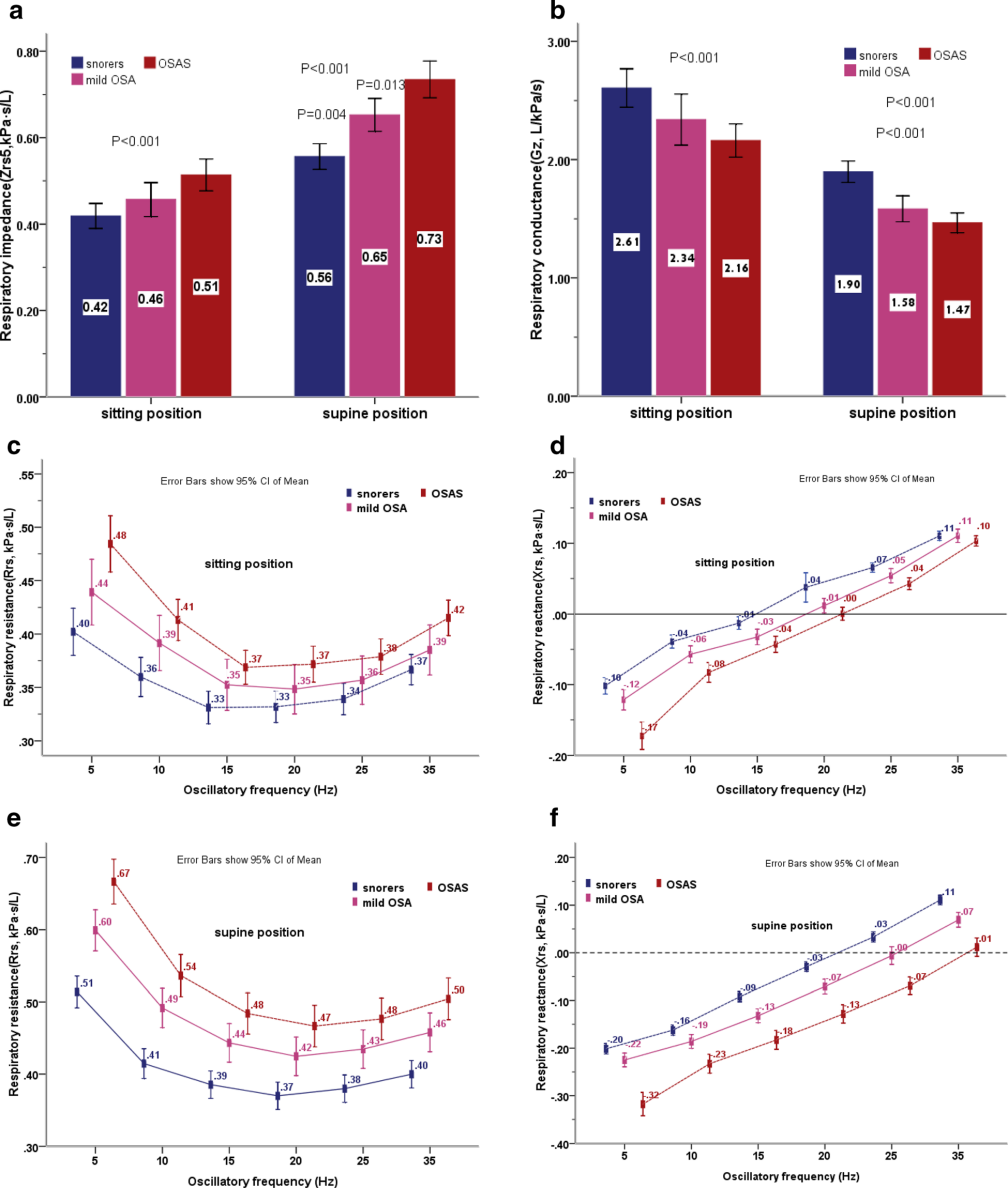
(**a**) Respiratory impedance at 5 Hz (Zrs5) in the sitting and supine position. Comparison was made using one-way ANOVA test. Significant differences in Zrs5 in the sitting position were found between the snorers and OSAS patients (*p* < .001), inter-group multiple comparisons were made using bonferroni method. Significant differences in Zrs5 in the supine position appeared between snorers and mild-OSA and OSAS patients. (snorers vs. mild-OSA, *p = .*004; snorers vs. OSAS, *p* < .001; mild-OSA vs. OSAS, *p = .*013). (**b**) Respiratory conductance calculated as the reciprocal of Zrs5, expressed as Gz. Comparison was made using one-way ANOVA test. Significant differences in Gz in the sitting position were found between the snorers and OSAS patients (*p* < .001), inter-group multiple comparisons were made using bonferroni method. Significant differences in Gz in the supine position appeared between snorers and mild-OSA, and OSAS patients. (snorers vs. mild-OSA, *p* < .001; snorers vs. OSAS, *p* < .001). (**c**) Respiratory resistance at different oscillatory frequencies measured by IOS in the sitting position. Comparison was made using one-way ANOVA test. Significant differences were found only between the snorers and OSAS patients (all *p* < .005), inter-group multiple comparisons were made using bonferroni method. (**d**) Respiratory reactance at various oscillatory frequencies in the sitting position. Comparison was made using one-way ANOVA test. Significant differences in X5 were found between the snorers and mild-OSA and OSAS patients (*p* < .003), inter-group multiple comparisons were made using bonferroni method. Significant differences in X10, X15, X20, X25were found only between the snorers and OSAS patients. (all *p* < .005) There was no significant difference for respiratory reactance at the 35 Hz frequency between three groups. (*p* = 0.419). (**e**) Respiratory resistance at different oscillatory frequencies measured by IOS in the supine position. Comparison was made using one-way ANOVA test. Significant differences in all frequency resistance were found between the snorers and mild-OSA, and OSAS patients, (*p* < .003) inter-group multiple comparisons were made using bonferroni method. There were no significant differences were found between mild-OSA and OSAS patients in the resistance from 10 Hz to 35 Hz. (**f**) Respiratory reactance at various oscillatory frequencies in the supine position. Comparison was made using one-way ANOVA test. Significant differences were found for all frequency reactance between the OSAS patients and mild-OSA, and the snorers (all *p* < .001), inter-group multiple comparisons were made using bonferroni method. There were no significant differences were found between the snorers and mild-OSA in the reactance at all frequencies

**Fig. 2:**
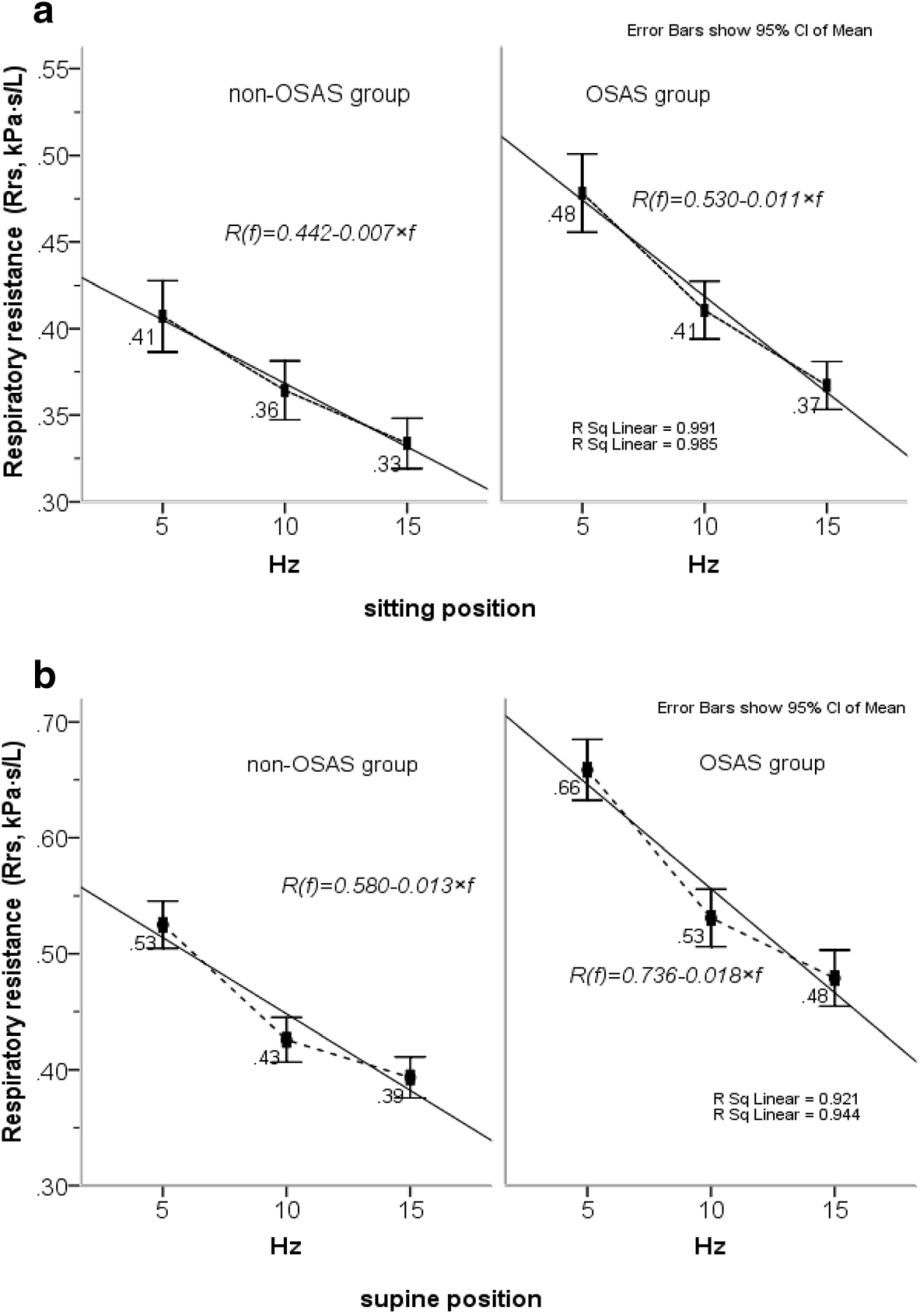
(**a**) Patients without and with OSAS. Rrs measured at low-frequency in the sitting position subjected to linear regression analysis of resistance vs frequency; Rrs at 0 Hz was back-extrapolating using equation of Rrs(f) = Rrs0 + S × f, (where f represents the frequency, S is the slope of the linear relationship of resistance versus frequency, Rrs0 is equivalent to zero-order frequency resistance, namely intercept), and then respiratory conductance at 0Hz(Grs) was obtained as reciprocal of Rrs0, which calculated for each subject in every condition according to linear model. The solid lines show the fit of linear regression mode. (**b**) Rrs measured at low-frequency (from 5 to 15 Hz) in the supine position subjected to linear regression analysis and resistance at the point of zero-frequency (Rrs0) was extrapolated using equation of Rrs(f) = Rrs0 + S × f . Grs in the supine position was obtained as the reciprocal of Rrs0, which calculated for each subject in every condition according to linear model. The solid lines show the fit of linear regression mode

In total, 230 patients who were preobese had a BMI 25.1 − 29.9 kg/m^2^ and obese had a BMI ⩾30 kg/m^2^ with normal lung function defined by FEV_1_/FVC >80 % of the predicted values were included in the study, while 16 patients were not included in analyses due to an absence of IOS data in the supine position. Anthropometric characteristics, lung function and volume data, and PSG data of all patients are summarized in Table 1. Of those 84 subjects were identified as healthy snorers whose RDI less than 5 events/h according to PSG results, 37 subjects with a RDI of 5–14 events/h were identified as mild–OSA, and 93 subjects with a RDI ⩾15 events/h were identified as OSAS patients. No significant differences were found between the three groups with respect to age, height, sex, FEV_1_/FVC, and lung volume data. There were significant differences in weight and BMI were existed only between healthy snorers and OSAS patients. The mechanical properties of the respiratory system measured by IOS for snorers, mild–OSA and OSAS patients in the sitting and the supine positions, are shown in Fig. 1a–f. Rrs0 was back-extrapolated separately, for each subject in each different condition, by applying linear regression equations of resistance vs frequency over the range of 5–15 Hz (Fig. 2a, b). The Rrs0 in the supine position were significantly higher with increasing severity of OSA as defined by RDI

**Table 1:** Anthropometric data and the respiratory function data of patients according to polysomnographic results

	snorers^[A]^	mild OSA^[B]^	OSAS^[C]^	*p* Value	*p* Value	*p* Value
	(*n* = 84)	(*n* = 37)	(*n* = 93)	[A] vs. [B]	[A] vs. [C]	[B] vs. [C]
Age, years	44.5 ± 8.4	45.6 ± 9.1	46.3 ± 10.9	1.000	0.708	1.000
Height, cm	166.7 ± 8.6	165.4 ± 8.3	167.2 ± 9.0	1.000	1.000	0.863
Weight, kg	79.9 ± 10.7	83.3 ± 14.1	88.4 ± 15.5	0.606	<0.001*	0.162
BMI, kg/m^2^	28.9 ± 3.3	30.4 ± 4.1	31.5 ± 4.3	0.150	<0.001*	0.393
Sex, male/female	57/27	23/14	72/21	0.160	0.160	0.160
FVC, L	3.81 ± 0.68	3.62 ± 0.67	3.77 ± 0.89	0.664	1.000	1.000
FEV_1_, L	3.08 ± 0.59	2.94 ± 0.55	3.05 ± 0.73	0.847	1.000	1.000
FEV_1_/FVC, % predicted	101 ± 6.0	103 ± 6.5	102 ± 6.2	0.394	0.858	1.000
TLC, L	5.70 ± 0.92	5.50 ± 0.87	5.68 ± 1.02	0.887	1.000	0.989
RV, L	2.02 ± 0.43	2.00 ± 0.37	2.06 ± 0.42	1.000	1.000	1.000
IC, L	2.29 ± 0.60	2.32 ± 0.61	2.51 ± 0.65	1.000	0.057	0.369
ERV, L	1.38 ± 0.54	1.17 ± 0.58	1.11 ± 0.54	0.171	0.004*	1.000
FRC, L	3.41 ± 0.67	3.17 ± 0.71	3.16 ± 0.71	0.267	0.062	1.000
Rrs0 seated, Pa · s · L^−1^	0.44 ± 0.13	0.49 ± 0.13	0.53 ± 0.16	0.234	<0.001*	0.340
Rrs0 supine, kPa · s · L^−1^	0.58 ± 0.12	0.68 ± 0.11	0.76 ± 0.18	0.004*	<0.001*	0.025*
Grs seated, L · kPa^−1^ · s^−1^	2.46 ± 0.66	2.19 ± 0.60	2.03 ± 0.56	0.067	<0.001*	0.580
Grs supine, L · kPa^−1^ · s^−1^	1.78 ± 0.34	1.51 ± 0.28	1.40 ± 0.33	<0.001*	<0.001*	0.195
RDI, events/h	2.60 ± 1.60	9.91 ± 3.78	41.75 ± 21.10	0.009*	<0.001*	<0.001*

*Abbreviations*: *BMI* body mass index, *FVC* forced vital capacity, *FEV*_*1*_ forced expiratory volume in 1 s, *FEV*_*1*_ */FVC, (% predicted)* ratio of FEV1 to FVC, (percentages of predicted values), *ERV* expiratory reserve volume, *FRC* functional residual capacity, *RV* residual volume, *TLC* total lung capacity, *IC* inspiratory capacity, *Rrs0* resistance at zero-frequency, is derived from linear-regression analysis of resistance vs frequency (over the 5 to 15Hz) and calculated for each subject in every condition according to the linear model (Fig. 2), *Grs* respiratory conductance at 0 Hz, the reciprocal of Rrs0, *RDI* respiratory disturbance index. The data are expressed as means ± standard deviation. Comparisons were made between groups using one-way ANOVA. *p*-values < 0.05 were considered statistically significant

* Exist significant difference ^[A]^ Snorers group ^[B]^ Mild OSA group ^[C] ^OSAS group

### Characteristics of IOS parameters in the sitting and supine positions in the snorers, mild–OSA and OSAS patients

As shown in Fig. 1a–f, Zrs5 and Rrs at all oscillatory-frequencies measured in the seated positions showed increasing trend to increasing severity of OSA . Zrs5 and Rrs at all oscillatory-frequencies were found significantly higher in the OSAS patients than in the snorers, and there were no significant differences between the mild-OSA and snorers, and mild-OSA vs. the OSAS patients. Obviously, this tendency appeared to increase when those patients moving from the sitting to supine position, especially, change in Zrs5 and R5 were notable, and significant differences were found between three groups. Conversely, Gz (the reciprocal of Zrs5) and Xrs at 5 Hz in the seated position were significantly lower in OSAS patients than mild–OSA, and than snorers. The same tendency mention above were also found in X5 in the supine position, This was significantly lower in OSAS patients than the both groups (mild–OSA and snorers). There were no significant differences were found between the snorers and mild-OSA in the reactance at all frequencies.

### Relationships between patient PSG outcomes and BMI, lung volume, and IOS measurements

Spearman’s rank correlation analysis was used to assess the association between IOS measurements in the both positions (sitting and supine) and RDI, and BMI (Table 2). BMI and Rrs, Zrs5 and R5 in the sitting position, were moderately positively correlated with RDI, while lung volume and Grs, Gz, and X5 were weakly to moderately negatively correlated with RDI. Those parameters also associated weakly to BMI, and the association was stronger when the IOS parameter was obtained in the supine position, as a close association was found between X5 measured in the supine position and RDI.

**Table 2:** Analysis of the relationships between PSG outcomes and BMI, lung volume, and IOS measurements in the sitting and supine positions

	RDI (events/h)	*p*-value (two-tailed)	BMI (kg/m^2^)	*p*-value (two-tailed)
BMI, kg/m^2^	0.39	0	-	-
Sitting position				
ERV, L	−0.221	0.001	−0.178	0.009
FRC, L	−0.141	0.042	−0.176	0.010
Rrs0, kPa · s · L^−1^	0.239	0.000	0.258	0.000
Zrs5, kPa · s · L^−1^	0.230	0.001	0.257	0.000
R5, kPa · s · L^−1^	0.217	0.001	0.255	0.000
X5, kPa · s · L^−1^	−0.379	0.000	−0.308	0.000
Grs, L · kPa^−1^ · s^−1^	−0.240	0.000	−0.260	0.000
Gz, L · kPa^−1^ · s^−1^	−0.230	0.001	−0.257	0.000
Supine position				
Rrs0, kPa · s · L^−1^	0.425	0.000	0.306	0.000
Zrs5, kPa · s · L^−1^	0.395	0.000	0.289	0.000
R5, kPa · s · L^−1^	0.378	0.000	0.298	0.000
X5, kPa · s · L^−1^	−0.517	0.000	−0.322	0.000
Grs, L · kPa^−1^ · s^−1^	−0.425	0.000	−0.305	0.000
Gz, L · kPa^−1^ · s^−1^	−0.395	0.000	−0.290	0.000

*Abbreviations*: *BMI* body mass index, *ERV* expiratory reserve volume, *FRC* functional residual capacity, *Rrs0* resistance at zero-frequency, *Zrs5* respiratory impedance at 5 Hz, *R5* respiratory resistance at 5 Hz, *X5* respiratory reactance at 5 Hz, *Grs* respiratory conductance, the reciprocal of Rrs0, *Gz* the reciprocal of Zrs5. All values are Spearman’s rank correlation coefficients (r)

### ROC of the IOS procedure in sitting and supine positions

**Fig. 3:**
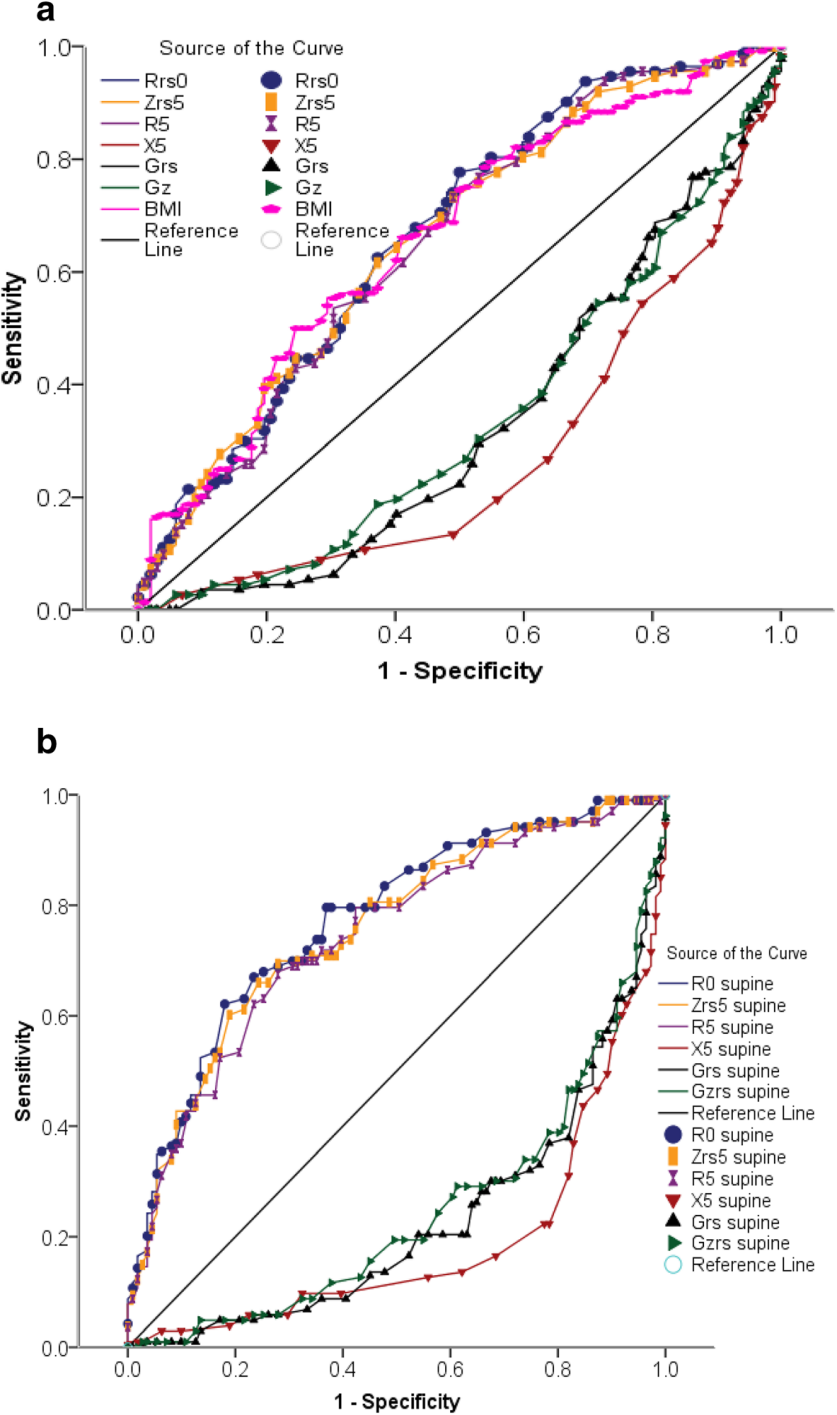
(**a**) ROC curves of the IOS parameters obtained in 93 patients suffering from OSAS and 121 non-OSAS patients in seated positions. The area under the ROC curve is a measure of the global ability of IOS parameter to correctly classify patients without and with OSAS (RDI < 15 or ⩾15/h). The value of the area under the ROC curve, respectively, for Rrs0 was 0.668 (95 % CI 0.596 to 0.740), for Zrs5 was 0.660 (95 % CI 0.587 to 0.732), R5 was 0.653 (95 % CI 0.580 to 0.727), BMI was 0.660 (95 % CI 0.588 to 0.733), Grs0 was 0.669 (95 % CI 0.596 to 0.741), Gz was 0.659 (95 % CI 0.587 to 0.732) and for X5 was 0.723 (95 % CI 0.655 to 0.792). (**b**) ROC curves of the IOS parameters obtained from all patients with and without OSAS in the supine position. The area under the ROC curve is a measure of the ability of IOS parameters to correctly classify patients of (RDI < 15 or ⩾15/h). The value of the area under the ROC curve for IOS parameters in the supine position, respectively, for Rrs0 was 0.772 (95 % CI 0.709 to 0.834), for Zrs was 0.754 (95 % CI 0.689 to 0.819), R5 was 0.743 (95 % CI 0.677 to 0.809), Grs was 0.772 (95 % CI 0.709 to 0.834), Gz was 0.754 (95 % CI 0.689 to 0.834) and for X5 was 0.811 (95 % CI 0.752 to 0.870)

The ability of the IOS parameter in the sitting and supine position to correctly classify non-OSAS patients including sroners and mild–OSA and OSAS patients (RDI <15 or ⩾15 /h) was estimated by calculating the area under the ROC curve (AUC; Fig. 3a, b). The values of the AUC for R5, Zrs5, Rrs0, their reciprocal Gz, and Grs ranged between 0.743 and 0.772 (95 % confidence interval [CI] range between 0.677 and 0.834), while X5 was 0.811 (95 % CI: 0.752–0.87) when the supine. When seated, the AUC values for these IOS parameters were globally lower, except for X5 (0.723, 95 % CI: 0.655–0.792). This indicates the potential usefulness of the values obtained in the supine position, but not in the seated position, for predicting OSAS.

From the ROC curve, the optimal cut-off values of the IOS parameters in the supine position were chosen as the point nearest to the top left corner to give maximal sensitivity and specificity. The cut-off values for R5, Zrs5, Rrs0, and X5 in the supine could identify OSAS patients among snorers and mild–OSA, with the best sensitivity and specificity. The positive predictive values (PPVs) and negative predictive values (NPVs) are given in Table 3.

**Table 3:** Sensitivity and specificity values used for detecting patients with and without OSAS, with PPVs and NPVs for the optimal cut-off points for the IOS parameters in the supine position

	Rrs0 (kPa · s · L^−1^)	Zrs5 (kPa · s · L^−1^)	R5 (kPa · s · L^−1^)	Grs (L · kPa^−1^ · s^−1^)	Gz (L · kPa^−1^ · s^−1^)	X5 supine (kPa · s · L^−1^)	X5 sitting (kPa · s · L^−1^)
Cut-off point	0.720	0.640	0.600	1.400	1.500	−0.23	−0.11
Se (%)	62.100	69.900	70.000	62.100	66.900	77.700	73.800
Sp (%)	82.900	72.100	72.100	82.000	72.100	79.400	62.200
PPV (%)	77.200	68.600	69.000	76.200	70.400	76.900	64.400
NPV (%)	70.200	71.600	70.200	70.000	72.100	80.000	71.900
LR+	3.630	2.505	2.590	3.450	2.400	3.770	1.950
LR−	0.457	0.417	0.416	0.462	0.459	0.281	0.421
Pre-test odds	0.920	0.920	0.920	0.920	0.920	0.920	0.920
Post-test probability (LR+)	0.810	0.700	0.706	0.760	0.688	0.776	0.648
Post-test probability (LR−)	0.296	0.278	0.277	0.298	0.297	0.206	0.279

*Abbreviations*: *Se* sensitivity, *Sp* specificity, *PPV* positive predictive value, *NPV* negative predictive value, *LR+* positive likelihood ratio, *LR−* negative likelihood ratio. The likelihood ratio is the likelihood that a given test result is expected in a patient with OSAS compared with the likelihood that the same result would be expected in a patient without OSAS, and calculated as sensitivity/(1 – specificity) for LR+, as (1 – sensitivity)/specificity for LR−, pre-test odds = P/(1-P), and post-test probability = pre-test odds*LR. Post-test probabilities for positive or negative tests were calculated according to simplified form of Bayes’ theorem

To identify which of IOS parameters in the supine position could best predicts the probability (*p*) of having a polysomnography positive for OSAS—the presence of OSAS (RDI ⩾15/h) or absence of OSAS (RDI <15/h)—we performed backward and forward stepwise logistic regression analysis using lung volume measurements, IOS parameters, and anthropometry as explanatory variables and the presence/absence of OSAS (yes/no) as dependent variables. In these regressions, the explanatory variables, such as Zrs5, Rrs0, and their reciprocals Gz and Grs, were not entered simultaneously into the equation:

$$ logit(p)= \ln \left(p/\left(1-p\right)\right)={\beta}_0+{\beta}_1{x}_1+{\beta}_2{x}_2+.....{\beta}_m{\beta}_m $$

Because these variables are highly dependent on each other, the correlation coefficients between Zrs5 and Rrs0, Grs, Gz were 0.961, −0.920, −0.939, respectively (all *p* < 0.0001). The logistic regression model results are shown in Table 4. The predictors Zrs5, R5, and X5 could independently and correctly classify patients with and without OSAS. Parameter of X5 obtained in the supine position singularly correctly classified 92 patients with a polysomnography RDI of <15 in the non-OSAS groups and 66 patients with a polysomnography RDI ⩾15 in the OSAS group. The first-step model construct using the forward stepwise method yielded a good specificity of 82.9 % and a poor sensitivity of 70.9 %. However, X5 combined with other explanatory variables (i.e. sex, Zrs5 and R5, or Rrs0 and Gz, or Grs) using the backward method entry into the regression model yielded a good sensitivity for identifying patients with OSAS. The highest of these was 75.2 % and the lowest 70.9 %, while the specificity remained relatively constant. We also constructed the same logistic regression model without explanatory variables of X5, but with explanatory variables of lung volume and anthropometry and combined these with the predictors Zrs5, Rrs0, Gz, or Grs. These models indicated that these predictors (Zrs5, Rrs0, Gz, and Grs) combined with BMI yielded a good sensitivity, but none were more specific than X5.

**Table 4:** Results of backward and forward stepwise logistic regression for predicting OSAS

Model construct by backward stepwise method	Explanatory variable	B	S.E.	Wald	*p*-values
First step	Weight	0.019	0.034	0.326	0.568
	BMI	0.067	0.119	0.314	0.575
	ERV	−0.643	0.553	1.354	0.245
	FRC	−0.067	0.526	0.016	0.899
	Sex	3.526	0.946	13.887	0.000
	Zrs5 supine	33.114	11.199	8.742	0.003
	R5 supine	−31.050	11.438	7.369	0.007
	X5 supine	−10.980	3.608	9.259	0.002
	Constant	−10.845	2.790	15.110	0.000
Last step	BMI	0.126	0.057	4.831	0.028
	ERV	−0.658	0.372	3.126	0.077
	Sex	3.761	0.847	19.743	0.000
	Zrs5 supine	33.474	11.223	8.937	0.003
	R5supine	−31.493	11.457	7.556	0.006
	X5supine	−10.814	3.560	9.229	0.002
	Constant	−11.356	2.282	22.774	0.000
Model construct by forward stepwise method					
First step	X5 supine	−15.475	2.650	34.094	0.000
	Constant	−3.797	0.627	36.654	0.000
Last step	Sex	3.559	0.849	17.572	0.000
	Zrs5 supine	34.100	11.483	8.819	0.003
	R5supine	−31.506	11.723	7.222	0.007
	X5supine	−11.372	3.583	10.076	0.002
	Constant	−12.428	2.240	30.784	0.000

*Abbreviations*: *BMI* body mass index, *ERV* expiratory reserve volume, *FRC* functional residual capacity, *Zrs5* respiratory impedance at 5 Hz, *R5* respiratory resistance at 5 Hz, *X5* respiratory reactance at 5 Hz. Outcome variable is the probability (*p*) of the presence of OSAS (RDI ⩾15) / absence of OSAS (RDI <15); namely, ln(*p*/(1-*p*)

## Discussion

In this study, we systematically appraised the ability of IOS parameters, obtained in supine and seated positions, compared with full polysomnography, for the detection of OSAS in preobese and obese snorers.

The parameter Rrs0, derived from linear-regression analysis of resistance versus frequency (over 5–15 Hz; Fig. 2a, b), its inverse Grs, and the IOS parameters at 5 Hz in the supine position, showed potential usefulness. We demonstrated that peripheral-airway parameter measurements by IOS in the sitting position were significantly and moderately correlated with the severity of OSAS as defined by RDI (Table 2). These associations were stronger when the IOS parameters were obtained in the supine position, and a highly significant association was found between X5 and RDI (*r* = −0.517, *P* = 0.000). The correlation coefficient between RDI and the peripheral-airway resistance parameters (R5), total respiratory resistance (Rrs0 and Zrs5), and respiratory conductance (Grs and Gz) were 0.378, 0.425, 0.395, −0.425, and −0.395, respectively, which are similar to results reported by Cao et al. in a previous study [22].

Shifting the posture from sitting to supine has been demonstrated to increase Rrs on FOT [23, 24]. This postural influence on respiratory mechanics has been attributed mainly to the effects of a reduction in lung volume [23–25]. Lung volume, in terms of FRC and ERV, significantly decreases when an obese subject adopts the supine posture due to a decreased outward recoil from the chest wall mass loading, as well as the gravitational effects of the abdominal contents. This results in a relaxed diaphragm that takes up a more expiratory position [26–28]. Because obese and obese OSAS patients already have a reduced FRC and ERV in erect and sitting postures, [15, 26] any further fall in FRC might lead to considerable airway closure or collapse. This could markedly increase airflow resistance in the intrathoracic airway, and may be more pronounced in the peripheral airways due to the lack of supporting structures such as the larger central airways. In the present study, there was a small decline in Rrs with increasing oscillation frequency between 5–15 Hz in the sitting position, which was exaggerated when our OSAS patients adopted the supine position. A striking rise in Rrs at 5 Hz was observed, exhibiting a more negative frequency dependence. In the non-OSAS groups (snorers and mild–OSA) there was little variation of Rrs with frequency and the Rrs at all frequencies was significantly lower than in the OSAS group in either the sitting or the supine position. Indeed, Rrs is roughly constant at lower-frequencies (from 4 to 16 Hz) on FOT in subjects with normal lungs, but shows a marked negative frequency dependence in patients with upper-airway obstruction and when the EELV was artificially lowered [14, 24]. Rrs at lower-frequencies in our OSAS patients presented a more negative frequency dependence than the non-OSAS groups. It was evident that most OSAS patients were experiencing low lung volumes, which may further decrease when those patients lie down, thereby contributing to a rise in Rrs at the serial site of the intrathoracic airways.

Other investigators have demonstrated that pharyngeal-airway collapsibility varies inversely with EELV. Reductions in EELV, by means of NEP or positive extrathoracic pressure on exhalation, causes decreases in caudal traction on the upper-airway and concomitant increases in upper-airway collapsibility, [29] presenting either with EFL or with airflow resistance increase in the upper-airway [6, 7, 30]. This is more pronounced in obese OSAS patients. The evidence implies that highly positive intrathoracic pressure, or driving pressure, would be generated when these patients begin expiration. Any additional pressure caused by NEP or positive extrathoracic pressure that favored the intrathoracic pressure or driving pressure to further evaluation, this may facilitate the collapse of the upper-airway as well as the early closure of small airways. Therefore, EFL induced by NEP is common in obese OSAS patients and can predict the severity of OSAS, as a high association between the degree of EFL measured in the supine position and AHI has been found [6, 7]. We found a remarkable significant increase in total respiratory resistance (Rrs0 and Zrs5) and a decrease in its inverse-respiratory conductance with increasing RDI in the OSAS group when they adopted a supine position. They also had a greater BMI than the non-OSAS groups. This may add evidence for the potential impact of obesity and postural changes on upper-airway patency and OSA susceptibility. Our results suggest that obesity, which substantially lowers EELV, can increase OSA susceptibility markedly by increasing pharyngeal collapsibility. This effect might be most marked when obese subjects sleep in the supine position, when EELV is the lowest [31].

Indeed, obesity has been proven to be the most common risk factor for OSAS. Obese supine subjects usually respire with a lower compliance of the lung and chest wall [32, 33]. Breathing with such a respiratory system determines larger changes in intrathoracic pressure, and even generates positive intrathoracic pressure surrounding the lung while relaxed at FRC level. Concomitantly, the high pleural pressure would cause tidal breathing to be initiated from low EELV where the lungs are less compliant and airways are prone to close on exhalation [34]. Breathing at lower end-expiration volumes has been demonstrated to significantly influence upper-airway patency, through loss of tension of caudal traction in the pharyngeal-airway, making it more folded and leading to increased pharyngeal collapsibility. In this study, the reactance (Xrs) at all oscillatory-frequencies measured in the supine position was found to be significantly decreased in the obese OSAS group. This decrease was remarkable for Xrs measured at 5 Hz (X5), and was found to strongly correlate with the severity of OSAS as defined by RDI and shows good predictive value for the diagnosis of OSAS in obese snorers. The X5 is a component of the out-of-phase air flow and pressure signal, and is numerically a negative value that reflects the sum elastance or compliance of the respiratory system. X5 values that are more negative indicate reduced respiratory system compliance or increased lung elasticity recoil pressure [14–16]. Our findings thus lead us to speculate that a narrowing of the upper-airway due to obesity causes inspiratory resistive breathing, and leads to respiratory compliance reduction in OSAS patients. This, in turn, may aggravate the upper-airway as well as peripheral-airway collapsibility, due to a loss of caudal traction tension on those structures at the end of expiration when the lung volume is lowest.

The ROC curves we constructed for Rrs0, Zrs5, R5, Grs, Gz, and X5 in both positions showed how these parameters can discriminate OSAS in preobese and obese snorers who are suspected of having this condition, with the diagnostic accuracy estimated by the AUC (Fig. 3). Only the parameters obtained in the supine position have an acceptable discriminating power making them suitable for clinical use. OSAS patients were optimally detected using the cut-off points of −0.23 kPa.s.L^−1^ for X5 with a good sensitivity 77.7 % and specificity of 79. 4 % and with a highly diagnostically accurate of 0.811 (95 % CI: 0.682–0.813). Among the predictors, X5 showed good PPV (76.9 %) and NPV (80 %) for the polysomnography diagnosis of OSAS.

A number of studies have used different and appealing approaches employing clinical and FOT parameters, or the degree of EFL, to predict the presence or absence of OSAS in suspected patients. Lorino et al. [30] reported that the FOT parameters of Rrs0 and Xrs at low-frequencies in the seated position were independently associated with the severity of OSAS. The ROC curves for those parameters were demonstrated to correctly discriminate patients with OSAS with sensitivities and specificities for Rrs0 and Xrs of 67 % and 93 %, and 90 % and 67 %, respectively. Zerah et al. [11] introduced that the parameter of sGrs in the seated position, combined with daytime oxygen-saturation, shows a 100 % NPV and a 86 % PPV with 100 % sensitivity and 84 % specificity for the diagnosis of OSAS in a large cohort of obese snorers. This suggests that obstruction in both structural upper and peripheral airways is common in obese OSAS patients. Previously, many studies have applied a small additional NEP to obese OSA patients in the sitting and supine, to induce EFL; these studies showed that the degree of EFL is a good predictor of this disorder [6, 7, 35–38]. The NEP technique is initially used for early detection of intrathoracic EFL in patients with obstructive lung disease who usually have lower lung compliance. The increased intrathoracic pressure gradients on exhalation caused by NEP facilitate the early closure of peripheral airways and result in expiratory flow decreased. EFL has been demonstrated to be common in obese OSAS patients when they experience NEP in the supine position, although the source of an EFL (intrathoracic or extrathoracic) from NEP has not been determined [35]. Our results are in line with Liistro et al. [35] and Verin et al. [6] who suggested that obese OSAS patients may undergo pharyngeal-airway and peripheral-airway collapse when sleeping supine, due to breathing at lower end-expiration volumes with less respiratory compliance and loss of caudal traction tension on those structures. In recent years, interest in the interpretation of Xrs parameters in obstructive disease has increased. Of particular clinical interest is Xrs measured at 5 Hz as a surrogate marker of EFL with NEP [39–42]. In the present study, we also used a statistical model based on lung function parameters in the supine position and combined this with anthropometry data to predict OSAS in preobese and obese snorers. This demonstrated that X5 combined with sex and ERV yields a good specificity (83.3 %) and sensitivity (76.8 %). Moreover, other predictors (Rrs0, Zrs5, and R5) combined with sex and BMI yielded acceptable sensitivities, but had lower specificity than X5 for predicting OSAS.

## Conclusions

In conclusion, the results of this study demonstrate that respiratory resistance parameters (Rrs0, Zrs5) and their inverses describing respiratory conductance (Grs and Gz), and the reactance parameter X5, measured in the supine position measured by IOS significantly correlate with the severity of OSAS as defined by RDI. Both the ROC curves we constructed and a statistical model based on daytime respiratory function abnormalities were effective for predicting OSAS in preobese and obese snorers who were suspected of having this condition. This study appears to indicate that the upper-airway, as well as the peripheral-airway, may experience obstruction or collapse simultaneously when OSAS patients are in the supine position as obesity increases the elastic load on these structures. Thus, reactance (X5) measurements are related to the degree of airflow obstruction, which had a moderate sensitivity and specificity for detecting OSAS. Therefore, IOS measurements might be useful as a screening test for OSAS during wakefulness.

### Limitations

One limitation of this research is that the study was carried out in subjects who attended a sleep clinic at a general hospital and, as such, are not typical of the population as a whole. In our study, the prevalence of OSAS was approximately 43 %, which is consistent with sleep clinic populations. The PPV and NPV of our index would likely be very different in the general population, where the prevalence of OSAS is reported to be 4 % in men and 2 % in women [43]. This bias effect is important because new diagnostic tests have an exaggerated diagnostic accuracy when evaluated in a sample with a limited disease spectrum. Thus, it should be emphasized that our results apply to the indications of sleep studies in patients referred to sleep clinics.

## Abbreviations

BMI: Body mass index
EELV: End-expiratory lung volume
EFL: Expiratory flow limitation
ERV: Expiratory reserve volume
FOT: Forced oscillation technique
FRC: Functional residual capacity
Grs: Respiratory conductance
IOS: Impulse oscillometry
NEP: Negative expiratory pressure
NPVs: Negative predictive values
OSA: Obstructive sleep apnea
OSAS: Obstructive sleep apnea syndrome
PPVs: Positive predictive values
RDI: Respiratory disturbance index
ROC: Receiver operating characteristics
Rrs: Respiratory resistance
Xrs: Respiratory reactance
Zrs: Respiratory impedance resistance.
